# Revealing functionally coherent subsets using a spectral clustering and an information integration approach

**DOI:** 10.1186/1752-0509-6-S3-S7

**Published:** 2012-12-17

**Authors:** Adam J Richards, John H Schwacke, Bärbel Rohrer, L Ashley Cowart, Xinghua Lu

**Affiliations:** 1 Department of Biochemistry and Molecular Biology, Medical University of South Carolina, Charleston, SC 29425, USA; 2 Department of Biostatistics and Bioinformatics, Duke University, Durham, NC 27705, USA; 3 Departments of Ophthalmology and Neurosciences, Medical University of South Carolina, Charleston, SC 29425, USA; 4 Department of Biomedical Informatics, University of Pittsburgh, Pittsburgh, PA 15232, USA

## Abstract

**Background:**

Contemporary high-throughput analyses often produce lengthy lists of genes or proteins. It is desirable to divide the genes into functionally coherent subsets for further investigation, by integrating heterogeneous information regarding the genes. Here we report a principled approach for managing and integrating multiple data sources within the framework of graph-spectrum analysis in order to identify coherent gene subsets.

**Results:**

We investigated several approaches to integrate information derived from different sources that reflect distinct aspects of gene functional relationships including: functional annotations of genes in the form of the Gene Ontology, co-mentioning of genes in the literature, and shared transcription factor binding sites among genes. Given a list of genes, we construct a graph containing the genes in each information space; then the graphs were kernel transformed so they could be integrated; finally functionally coherent subsets were identified using a spectral clustering algorithm. In a series of simulation experiments, known functionally coherent gene sets were mixed and recovered using our approach.

**Conclusions:**

The results indicate that spectral clustering approaches are capable of recovering coherent gene modules even under noisy conditions, and that information integration serves to further enhance this capability. When applied to a real-world data set, our methods revealed biologically sensible modules, and highlighted the importance of information integration. The implementation of the statistical model is provided under the GNU general public license, as an installable Python module, at: http://code.google.com/p/spectralmix.

## Background

In biomedical sciences, experimental results often come in the form of one or more gene sets, and biologists are commonly tasked with the interpretation of these lists, which can easily become overwhelming considering the amount of data and number of data sources currently available. Frequently, gene products carry out their function by working closely with the products of other genes, which motivates the study of genes as a set, instead of as individual units. We refer to these multi-gene units when carrying out one or more related biological processes as 'functional modules'. There are a number of rationales for studying genes through a modular perspective [1-3]. Modules of genes may be interesting because of physical interactions [4], common subcellular location [5], or they may be meaningful players in a system of interconnected biologically processes. Whichever the case, it is of significant interest to be able to hone in on interesting subsets of genes [6] that perform coherent functions, particularly by making use of multiple types of information sources [7].

Currently, a common approach to discovering functional modules from a gene list is via the use of enrichment-based methods [8-10], which determine if constituents of a predefined collection of gene sets are observed more frequently than expected in the list. Often, these predefined reference gene sets reflect a single information source; for example, gene sets are commonly grouped according to annotations based on the Gene Ontology (GO) to narrow the search based on one or more known functions. The requirement of predefined gene sets subjects the methods to limits imposed by those who construct the gene sets, thus reducing the chances of finding *de novo *coherent subsets. In situations where the nature of the interesting subsets is unknown, data-driven methods are more suitable than methods based on predefined reference sets. Additionally, because evidence for gene-gene relationships within a module may occur in different forms, a caveat of most existing methods is that they do not consider the connections across distinct biological aspects, and thus would fail to identify diverse types of functional modules.

Experimental methods and thus their resulting data come in many diverse forms, and in light of this it remains challenging to assess the functional coherence of a group of genes by considering multiple biological aspects. As an example, consider the following hypothetical scenario: from protein-protein interaction data, we find that protein *A *physically interacts with protein *B*, and from a signal transduction database one learns that protein *B *is a kinase that phosphorylates protein *C*. The challenge is to find out that proteins *A*, *B*, and *C *are functionally related in an automated way. Here, we describe a novel approach for revealing functionally coherent subsets *ab initio *from an arbitrary gene list by assimilating information from multiple data sources.

There are two main challenges with combining heterogeneous information to identify functionally coherent subsets from a gene list. First, storing and accessing multiple information sources can be challenging for organisms of modest to large genome size, for which we implemented a web server to handle storage and facilitate access (See Supplemental Methods in the Additional File 1). Second, it remains an active research area to encode diverse information regarding genes in a fashion that enables identification of functional modules. One notable method [11] that uses a Bayesian approach to integrate heterogeneous data sources was devised for the purposes of function prediction. The problem of identifying functionally coherent subgroups is a related but distinct problem to that of function prediction.

**Figure 1:**
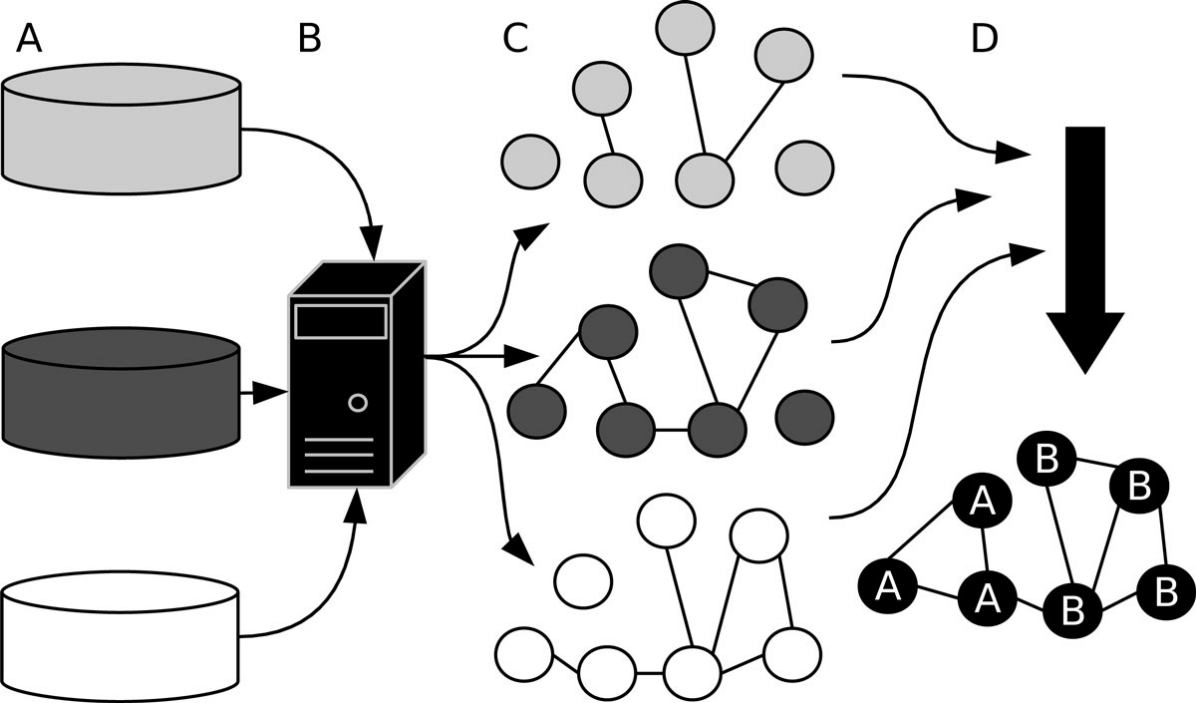
**Conceptual diagram of analysis pipeline**. In this example, information is combined to partition seven genes. **A **Input gene-gene relationships are gathered from databases or via other approaches, like experimentation. **B **A web server is used to store, organize and provide programmatic access to the data. **C **Given a gene list of interest, graphs are constructed with connections among the genes representing gene-gene information for a given information source. **D **Graph edge weights are transformed into kernel space using gene-gene distances. The affinity-weighted graphs are combined into a summarizing graph and subsequently the summarizing affinities are projected into eigen-decomposition space, where the genes are partitioned.

In this study, we represented genes as nodes of a graph whose edges reflect functional relatedness among the genes, based on available information regarding genes. Then the task of identifying coherent gene subsets is reduced to the task of finding highly connected subgraphs from the graph, for which different existing graph-cut algorithms can be employed. Finally, it is non-trivial to integrate information from multiple sources in a unified manner such that coherence of the gene subset is assessed utilizing all information. This is particularly important because information from each source can be limited but complementary to each other, thus combining them can potentially enhance the overall performance of revealing coherent subsets. To this end, we propose to use a spectral projection approach, described in Figure 1, in which relationships between genes are represented as graphs in different information spaces (sources) and are further combined in kernel-transformed space. Under such a setting, coherence subsets were further refined using spectral clustering methods. This work represents the following methodological contributions: First, we show that functional modules can be recovered by applying a graph-cut algorithm, presented as a form of the spectral clustering algorithm originally introduced by Ng, Jordan and Weiss [12]. Second, we demonstrate that combining data in kernel-transformed space enhances the ability of the algorithm to recover gene sets. Additionally, the method is recast in a way to account for the potentially large percentages of noise present in gene lists coming from experiments. Finally, an application of the approach to gene expression data [13] revealed that many of the uncovered gene subsets were biologically sensible in that they belong to appropriate biological processes.

## Results and discussion

In this section, we detail the results of a number of simulation experiments as well as an example application. Using a simulation approach, we examined the algorithm's ability to retrieve gene subsets, and specifically, we studied how the addition of new data sources impacts performance. Then, we tested the usefulness of our approach in recovering coherent gene set from 'noisy' gene lists as is often encountered in high-throughput experiments. We then show the results of applying our method to a real-world data set.

### Discrimination of gene sets by spectral clustering

Given a gene list, our task is to identify functionally coherent gene subsets. Here, we used simulation experiments to evaluate the efficacy of spectral clustering for this task. In the simulation experiments, a number of functionally coherent gene subsets, ranging from 3-8, was randomly mixed in multiple experiments, and our method was then used to recover the original subset partitions. We used pathways from KEGG database [14] and protein complexes from the MINT [15] database as 'known' functionally coherent modules, in that the proteins in these modules either perform related functions or form physical modules.

**Figure 2:**
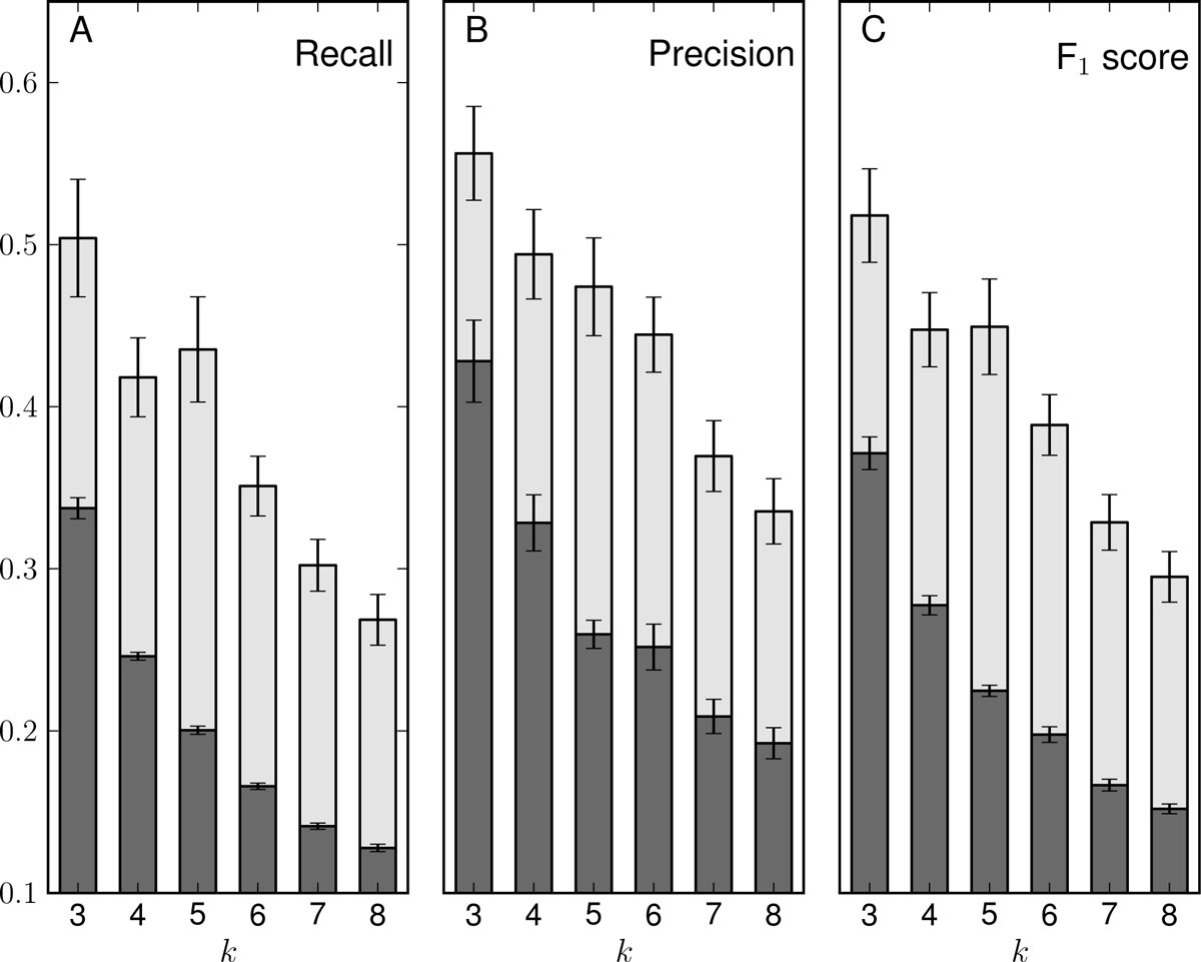
**Evaluating the algorithm's discriminative abilities**. Using simulations and spectral clustering as described in the methods, algorithm performance is summarized by recall (**A**), precision (**B**) and F1 scores (**C**) as a function of increasing *k*. Each bar represents an average of 20 simulations, and for each value of *k *the same data and cluster assignments were used to find the shown recall, precision, and F_1 _score. The dark gray portions of each bar are the results if cluster assignments were randomly guessed. For these simulations the organism used was *S*. *cerevisiae*, and the information source was the Gene Ontology. Standard error bars are included.

We performed a series of experiments by mixing an increasing number of 'known' functionally coherent gene sets, *k*, which was repeated with the given *k *20 times. The experiments were carried out using the GO annotations as the information source for the algorithm. The results in terms of recall, precision and F_1 _scores are summarized for *S. cerevisiae *in Figure 2. Also, shown in the figure are the results of randomly assigned cluster labels as a control experiment.

The figure shows that the spectral clustering algorithm significantly outperforms random cluster assignments, and the difference becomes more obvious as *k *increases. Overall, the trend for spectral clustering is that of decreasing efficacy with increasing *k*, and in the case of the GO precision and recall, both are similarly affected. This decreasing trend is likely due to the fact that in general, clustering tasks become more difficult as more gene sets are mixed. An additional reason for the declining performance might be the fact that many metabolic and signal transduction pathways, as well as molecular complexes, are comprised of a mixture of functional modules, or coherent gene sets, and thus assigning modules to an appropriate pathway is not a straightforward task, especially as the number of modules and potential pathways increases.

### Information integration to enhance clustering

**Figure 3:**
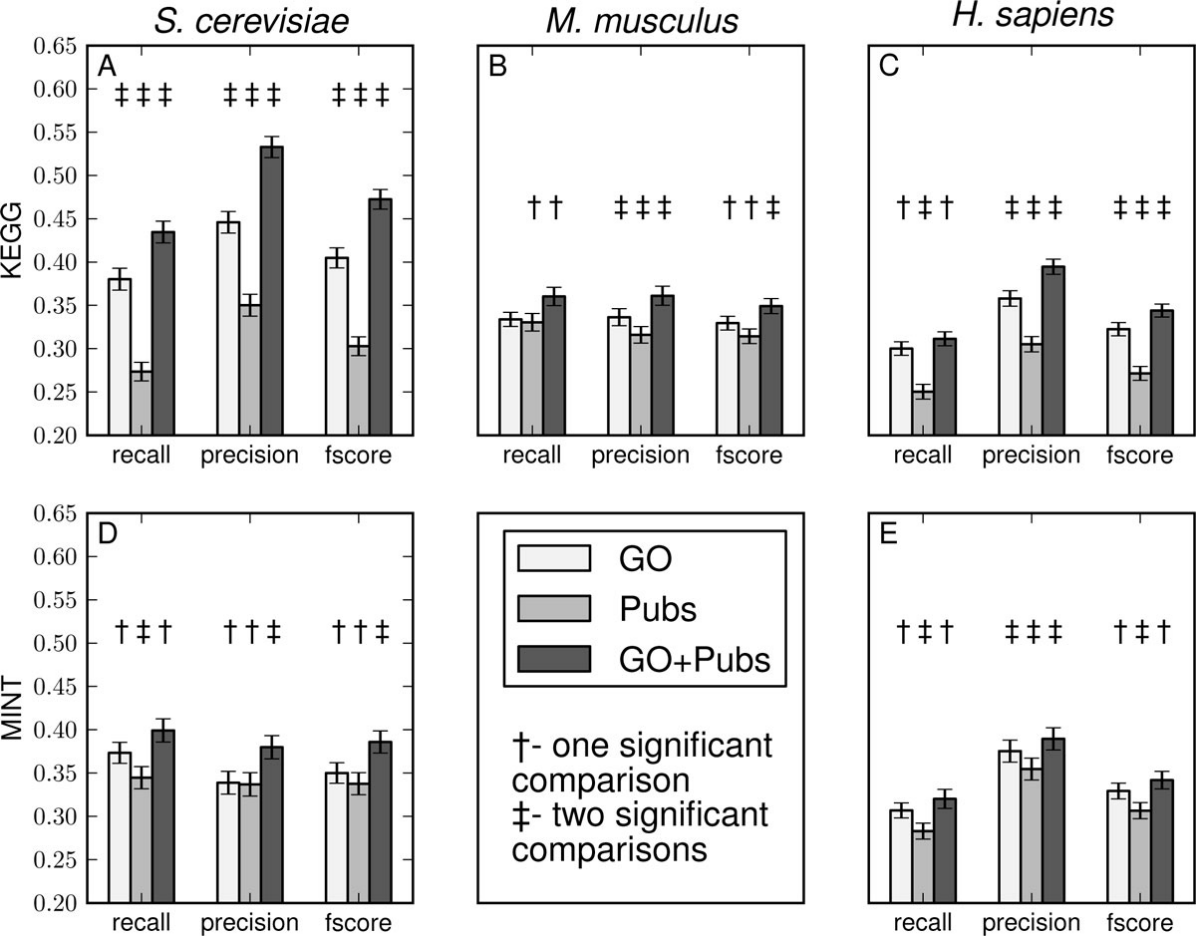
**Literature and Gene Ontology integration**. The discriminative abilities as represented by recall, precision and F_1 _scores, are shown for the Gene Ontology, PubMed and combined simulations. Each subplot from left to right is a summary of 120 individual simulations for the species *S. cerevisiae*, *M. musculus*, and *H. sapiens*, respectively. The rows correspond to simulations run with the KEGG and MINT positive control modules. Each set of 120 simulations was comprised of a mixture of individual runs, where the number of pathways ranged from 3-8. Standard error bars are given for each discriminative measure, and for each of the three species. Significance was tested for across the data source combinations for each evaluator independently.

We then tested if information integration would further enhance the algorithm's ability to correctly partition mixtures of genes. We used data from the Gene Ontology (GO) and PubMed databases as the information sources for this experiment. We also considered the information pertaining to different species: *Saccharomyces cerevisiae*, *Mus musculus *and *Homo sapiens*, in order to test the generalizability of spectral clustering and information integration in this context. A total of 120 simulations were run for each species and each combination of information sources, using both KEGG and MINT as positive controls. The results are summarized in Figure 3. If we use average F_1 _scores to summarize the performance under all simulations, the spectral clustering algorithm performed better in each case when data were combined than for either single-source version. In general, simulations carried out on *S. cerevisiae *had greater precision and recall than those done on *H. sapiens *and *M. musculus*. The pattern is likely due to the fact that the higher eukaryotes have larger genomes, and correspondingly, the information available is sparser when compared to the well-studied and genomically smaller Baker's yeast. Each bar is an average of 120 simulations ranging in specified *k *from 3-8. We also observed that the GO alone has better precision than the literature alone for all species studied, and the combination of GO and publications consistently performs the best of the information sources.

The observed improvements in data partitioning due to information integration is highly encouraging. The results indicate that indeed, different information sources contain distinct yet complementary information, and efficient information integration techniques can be employed to utilize such complementary information in order to achieve a better gene set recovery. The kernel fusion and transformation step in spectral clustering (see equations 3, 4, and 5) provided a principled way of integrating information in that the sum of two kernel functions does not require exceptions and heuristics.

Our results show that spectral clustering performs better using the GO as an information source in comparison to gene co-mentioning data (PubMed). One possible explanation is that information from the GO database is 'richer' in comparison to that of the gene co-mentioning. It is easier to establish the relatedness among a pair of genes in terms of function because many genes are annotated in the GO databases, and our approach of revealing functional relationships using the graphical representation of the GO can easily assess the relatedness between a pair of genes, even though they may be annotated with different GO terms. We believe the strength of our approach lies in the fact that it captures the functional relationship between genes, by taking into account both the structure of the GO and the strength of the relationship using semantic distance. This observation may lead to other possible approaches of representing the functional relationship between genes; for example one could use rigorous topic modeling of literature information that are associated with a gene in order to capture the functional relationships between genes [16,17]. On the other hand, the gene co-mentioning data matrix is fairly sparse, and not all information is directly relevant; thus, as an information source alone, gene co-mentioning does not perform well. Finally, a key observation from this experiment is that, although an information source may not be rich in information, it may be valuable if it is complementary to other information sources.

### Discovering modules amongst noise

**Figure 4:**
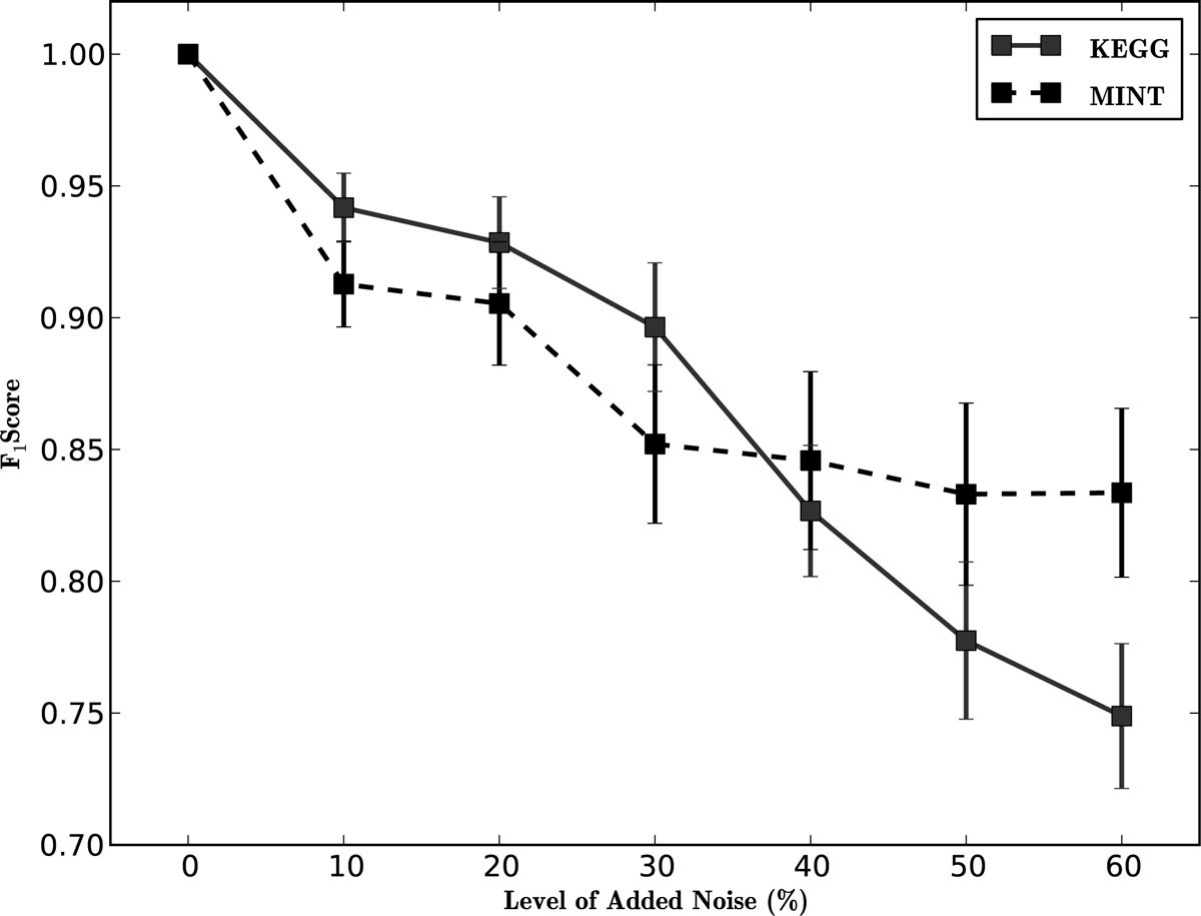
**Recovering functional modules contaminated with noise**. The original gene lists for KEGG and MINT (*N *= 22 and *N *= 24 respectively) were compiled and increasing levels of noise were added to each set, where each was then considered a new list. Spectral clustering was run on each gene list and statistical significance based on functional coherence was determined for all the the underlying modules. Statistically significant modules were assembled and together they made up the positively labeled genes. Shown here are the averaged results of the gene sets, with standard error bars at each level of noise.

Biologists and experimentalists are accustomed to working under the assumption that there is at least a minimal level of stochastic fluctuations in the accuracy of experimental results. A key for analyzing biological experimental results is to identify real signal from within the 'noisy' data; in our case, to recover coherent gene sets from data contaminated with noise. In this experiment, we used MINT and KEGG derived gene sets (*S. cerevisiae*), and we injected genes randomly selected from the genome to serve as noise. The experiment was repeated at seven levels of noise for 24 KEGG and 22 MINT gene sets. The goal was to run the algorithm, without making an assumption for *k*, and check the ability to recover the original coherent subsets. Using combined GO and PubMed data as information sources, our algorithm broke each gene set into *k *clusters for which each was then subjected to coherence testing using the GOSteiner method [18]. Only statistically significant modules were considered coherent. Based on observed F_1 _scores in Figure 4 we can summarize that our procedure identified correct genes, while minimizing false positives for minimal levels of noise contamination (0-20%). At higher levels of noise the average performance among gene sets drops to less desirable levels. The pattern for KEGG and MINT gene sets is comparable, with MINT performing slightly better at high (≥50%) levels of noise. The levels of injected noise are not the realized amount of noise, which are generally a few percentage points lower, due to the fact that not all genes have information with respect to the data sources. Overall, this is an impressive result indicating that the techniques in our procedure (spectral clustering, information integration, determination of cluster number, and finally functional coherence assessment) successfully revealed the majority of the truly coherent genes even at relatively high levels of noise.

To further evaluate the results, we plotted the individual simulations as graphs, and inspected the calls made by the algorithm (see Supplemental Results in the Additional File 1). From these observations, we see that, in general, the false positive genes are weakly connected to the rest of the true positive genes, which explains why they were not included as part of the noise group. Also, the true positive genes are generally highly connected, in terms of edge weights, which indicates that spectral clustering is capable of accurately capturing the relatedness of functionally coherent genes, and our overall procedure is capable of dealing with noise inevitably found in biological data.

### Application to gene expression data

**Figure 5:**
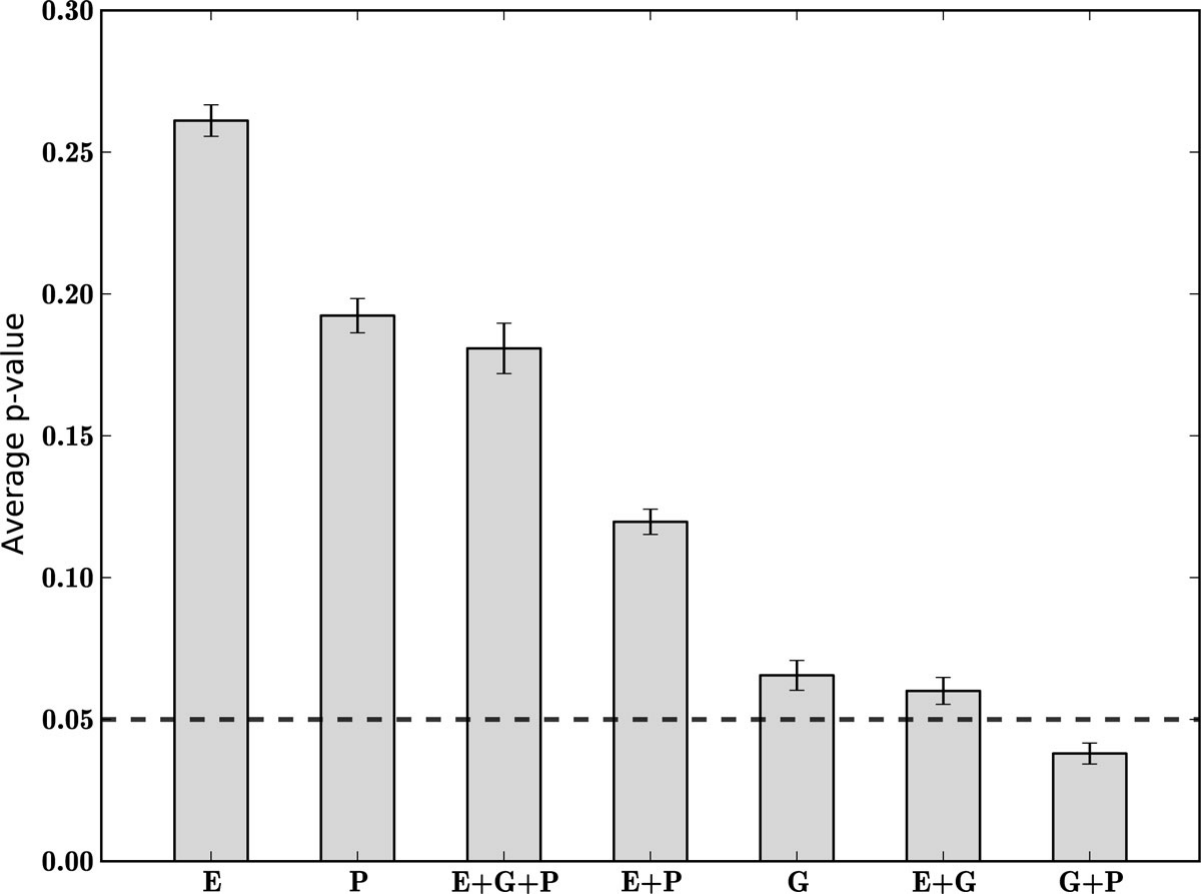
**Application to gene expression example**. The genes of interest (458 total) were partitioned according to one or more information sources and the resulting subsets were assessed for functional significance. Each bar designates an average of p-values for all 458 genes based on a unique partitioning of the genes using one or more information sources. The publications (P), Gene Ontology (G), gene expression (E), and combinations of each are shown. The standard error bars for the averaged p-value are shown for each clustering result and the traditional level of *α *= 0.05 is shown for reference.

The proceeding sections provided both theoretical and empirical rationales for using the proposed information integration framework to mine large gene lists for functional modules. To test the method with a real-world data set, we chose to use a gene expression data set that is well-studied [13,18,19]. The animal model is the *rd1 *mouse, which is a commonly used model [20] for *retinitis pigmentosa *(RP), a disease characterized by rod photoreceptor degeneration and apoptosis [21]. Recent work [22] provided guidance in creating the gene set of interest (458 genes). Here, we tested the ability of our procedure to identify functionally coherent subsets from this real-world data set, which is within the range of small to moderately sized gene sets (*N <*1000) often observed in experiments. The results are summarized in Figure 5. For each information source combination, the filtered set of genes was partitioned using spectral clustering and each subset was subsequently tested for statistical significance using GOSteiner [18]. The results are summarized using a weighted average of p-values with the p-value for a given gene corresponding to that of its assigned cluster. All combinations of data sources containing the gene expression information source arranged the genes in to less functionally coherent gene sets when compared to the other information sources. Gene expression data likely does not provide sufficient information regarding the function of genes, based on the current state of data sources and hence we do not suggest including it unless the study is exploratory in nature. It is possible that gene expression derived gene sets are quite meaningful functionally, however it is difficult to evaluate uncharacterized gene interactions. Interestingly, the GO alone is not the best performing, which provides additional support for the use of publications as an additional data source. For the publications alone, we already know from the simulation studies (see Figure 3) that we cannot expect robust subset discovery, however in combination with other sources the effect appears to be additive.

## Conclusions

Overall, the methods presented in this paper allow for efficient gene subset searching in both simulated and the real-world data. Our approach should be of interest to a spectrum of biologists: it can be used to sift through large amounts of experimental data, and will help the experimentalist to identify specific genes or biological functions of interest. A method that effectively partitions mixtures of genes into functional modules is highly desirable in contemporary high throughput biology, particularly in microarray studies. Our results show the value of spectral clustering, and particularly information integration in this setting. This research also prompts new research avenues, including: the discovery of additional informative data sources, and the adaption of these techniques to other problems like the prediction of gene function.

## Methods

### Sources of information

#### GO: an information source of functional relationships between genes

The GO defines the relationships between annotation terms in a hierarchical way, using expert knowledge. Annotation and ontology definition files used in this study were downloaded from: http://www.geneontology.org/GO.downloads.database.shtml (03.16.2011). Given the ontology structure and annotation information, a variety of methods and information sources have been proposed to quantitatively describe the relationships between terms [23,24], often referred to as semantic distances. The distances were used to construct a weighted graph of all terms provided by the GO. The GO graph was then used to quantify the distance for any two genes. Edges were not drawn when the following evidence codes were used: Inferred from Electronic Annotation (IEA), Inferred from Sequence or Structural Similarity (ISS), Inferred from Sequence Orthology (ISO), Inferred from Sequence Alignment (ISA), Inferred from Sequence Model (ISM), Inferred from Genomic Context (IGC), and inferred from Reviewed Computational Analysis (RCA).

Lets consider the GO as the singular data source so that a graph *G *= {**V**, **E**, **w**} may be defined such that all gene vertices **V **are connected using a set of edges **E**. The edges are then given weights **w **reflecting the semantic distance. To quantify the relationships between terms, a relative difference in information content (IC) may be used [25-28] as a measure of semantic distance. Adopting this principle, the IC of a GO term is calculated as follows:

(1) IC(t)=-InP(t),

where *P*(*t*) is the number of annotation instances for the term divided by total number of instances from the annotation database. We can then define the semantic distance between a parent-child pair of GO terms as

(2) $$\text{dist}\;\left(t_{p},t_{c}\right)=|\text{IC}\left(t_{p}\right)-\text{IC}\left(t_{c}\right)|$$

The goal of constructing a weighted graph representing the structure and semantic relationships of the GO is to use this data structure to determine the functional relatedness of genes, because a pairwise distance matrix among the genes is needed for spectral clustering. For each gene pair, all GO terms that were used to annotate the two genes were considered, and functional distance between the genes was may be determined as the distance of the shortest path between the genes in the GO graph, using a bidirectional version of Dijkstra's algorithm [29] as implemented using NetworkX [30]. Using the information integration techniques discussed below, the three aspects of the GO: *biological process*, *molecular function *and *cellular component *were combined. The distances between genes tend to be smaller for cellular component than for the other aspects (see Supplemental Results in the Additional File 1). However, by using all three aspects simultaneously a single very small distance will have less of an affect on overall gene-gene distance than three reasonably small distances.

#### Gene co-mentioning: an information source of relatedness among genes

When a pair of genes is co-mentioned in the biomedical literature, they are often related to each other somehow: they may be participating in the same biological processes co-operatively or, alternatively, they may counter-act each other. The reasons for the co-mentioning are many; nonetheless, a biomedical document seldom mentions genes that are totally irrelevant, although certain exceptions exist. To populate a pairwise distance matrix of all genes using co-mentioning data, a current file containing a mapping between genes and biomedical literature was downloaded from NCBI FTP site ftp://ftp.ncbi.nlm.nih.gov/gene/DATA/gene2pubmed.gz. Using these data, the distance between two genes was calculated as the maximum number of shared publications minus the observed number of shared publications.

### Spectral clustering for gene list partitioning

Spectral clustering aims to divide a set of data points into highly related subsets. Unlike conventional clustering methods such as *K*-means clustering, spectral clustering groups data points based on their 'relatedness' rather than their geometric closeness. As a result, a set of data points can be partitioned into a cluster based on a chain of strong pairwise connections even though the points are geometrically remote. Thus, the method is particularly well-suited for capturing the relationships between gene subsets by taking into account their relatedness across different biological aspects, for example, gene products that are linearly connected in a metabolic pathway.

Given a list of genes, pairwise relationships were used to construct a distance matrix that was subsequently used as input into the version of spectral clustering proposed by Ng and colleagues [12]. The first step of the algorithm is to transform the distance matrix into an affinity matrix using a kernel. In this context, the kernel functions to provide a means by which the pairwise relationships between genes are modulated; an essential ability when combining data that vary in distribution, shape and range. Affinities were calculated using the Gaussian kernel function:

(3) $$A_{ij}=\exp\left(\frac{-{\left||d_{ij}|\right|}^{2}}{2\sigma^{2}}\right),$$

where ||*d_ij_*|| is a measure of distance between objects *i *and *j *and *σ *is the bandwidth parameter. In related works, *σ *was automatically scanned for by minimizing a quantity referred to as *distortion *[12,31], an objective function that assesses the quality of the clustering. Empirically, we have found that searching for *σ *based on distortion tends towards increasing recall at the expense of precision; therefore we opted to search for an optimal *σ *by maximizing the mean silhouette value [32] instead. A silhouette value measures how similar to each other the data points in a cluster are, relative to the points outside the cluster, and thus reflects the coherence of a cluster. The values of *σ *used in this study are reported in Supplemental Table 1 in the Additional File 1. The parameters were estimated by mixing groups of known functionally coherent groups, scanning intervals of possible values, calculating precision and recall, and finally by visually inspecting both the affinity values as well as the plotted results.

Here, we formalize the algorithm steps described so far, and describe the remaining parts. Given a set of genes **g **= {*g*_1_, ..., *g_N_*}, a distance matrix is first constructed, which is further transformed into an *affinity matrix ***A **in which an element, *a_ij_*, is determined by Eqn 3. Next, a *diagonal matrix ***D **is then created such that the (*i*, *i*)-element is the sum of **A**'s *i*-th row. With these data, the *Laplacian matrix ***L **may then be computed.

(4) $$\text{L}={\text{D}}^{-1/2}\text{A}{\text{D}}^{-1/2}$$

Next, we find *x*_1_, ..., *x_k_*, which are the *k *eigenvectors associated with the *k *largest eigenvalues of **L**, and form the matrix **X **= [*x*_1_, ..., *x_n_*] by stacking the eigenvectors horizontally. Then, a normalized matrix **Y **is found by transforming each of the rows in **X **to have unit length. For example,

(5) $$y_{ij}=\frac{x_{ij}}{{\left(\sum_{j}x_{ij}^{2}\right)}^{1/2}}$$

Treating each row in **Y **as a point, the points are then clustered into *k *subsets using the *K*-means clustering algorithm. Finally, the original point *g_i _*is assigned to cluster *j *if row *i *of the matrix **Y **was assigned to cluster *j*. We note that *L *is not actually the Laplacian (*I - L*) as traditionally thought of from graph theory, though we keep with the terminology of Ng *et al. *[12]. To carry out the clustering and related tasks, an installable Python package was developed and made publicly available through a mercurial repository http://code.google.com/p/spectralmix.

### Integrating heterogeneous data

One of the major goals, given a gene set of interest, is to integrate the information from distinct information sources, such that one can take advantage of complementary information to reveal the connections among genes that would be missed when an individual information source is used. Within the framework of spectral clustering, information integration can be performed at different stages: 1) create a pairwise distance matrix by combining all information sources, 2) after kernel transformation, combine the similarity matrices derived from different information sources in the kernel space. Integration at the distance stage is inherently difficult, because of differences in location, scale, and distribution types of distinct sources. The second approach is also referred to as a kernel fusion approach [33], which is not only a logical approach to integrate data, but is also shown to be effective. A major advantage of this approach is that it is principled, that is the same approach is taken each time, thus avoiding the issue of technique manipulation for newly encountered information sources. In this study, we performed kernel transformations of distance matrices from each information source into corresponding affinity matrices, which were scaled using an information-source-specific *σ*. Then, affinity matrices were element-wise summed to produce a unified affinity matrix.

### Identifying coherent gene subsets

Just because we have a set of genes partitioned into groups does not necessarily mean that the resulting clusters will represent coherent gene subsets; for this there are three challenges that must be overcome. The first is that of noise: experimental results commonly contain noise and as a result any method that clusters genes must be shown to be reasonably resilient to random noise. The second is determining the optimal number of subsets; and the final challenge is to assess whether a subset is functionally coherent or not. To judge the quality of a given clustering, the average silhouette value for a cluster may be used, where values ≤ 0 are considered poorly clustered. This heuristic is useful for filtering or ranking, however it does not tell us much about method performance in the face of noise. In order to determine the extent to which noise plays a role a more rigorous set of simulations was run, where known positive control data sets were combined with varying quantities of inserted noise.

In order to determine the suitable number of clusters to partition data another modification was made. Given data **X **that have been kernel transformed and cast into eigen-decomposition space as **Y**, we consider the first two eigenvectors. Originally, **Y **may be partitioned in this space using *K*-means or another clustering algorithm, however we may repartition the data by scanning over a range of *k *(3-8) settling on the value that maximizes the average silhouette index [32]. Because *k *is normally unknown, the search for an optimal number of clusters is necessary component of the algorithm.

The resulting *k *subsets were then analyzed for functional coherence [18]. The method described therein, also called GOSteiner, is based solely on the Gene Ontology and uses a graph-theoretic method to determine statistical significance, in terms of functional coherence, of an arbitrary gene set. Given the current state of functional annotation completeness for the GO, it is expected that there are some number of functionally interesting clusters that will be missed, however the number of false positives is expected to be very low with GOSteiner.

### Simulation and evaluation

The simulations experiments provide a controlled environment to serve as a common means, by which comparisons can be made over a variety of experimental conditions including: the impact of different approaches for populating and calculating distance matrices, the effect of combinations of different data types, and the impact of noise on clustering. In this study a varying number (*k*) of functionally coherent gene sets were randomly selected, mixed, and combined to form a single gene set. The functionally coherent sets come from the Kyoto Encyclopedia of Genes and Genomes (KEGG) database [14] and the Molecular INTeraction (MINT) database [15]. The clustering algorithm was then applied to partition the genes. For each iteration of the simulation to a newly created gene set, it is necessary to evaluate the clustering assignments. We used an evaluation method that counts pairs of similar and non-similarly labeled genes in the same way the Rand index [34] is calculated. This evaluation method allows the calculation of both precision and recall and a summarizing F_1 _score (see Supplemental Methods in the Additional File 1). It is important to note that precision and recall as traditionally thought of in information retrieval is different from this setting, because we are considering pairwise relationships instead of the genes themselves. Simulations were run 20 times for each *k*, in order to carry out performance comparisons under different conditions; for example, to compare the use of different information sources. With the simulations run, the data were grouped based on the simulation condition and performance metric. In order to statistically compare these groups, normally an ANOVA would be used. However, ANOVA with repeated measures could not be used to compare the groups or blocks (e.g. GO, Pubs, GO-Pubs), because the assumptions of equal variance and normality were violated. To check the model assumptions, the Shapiro-Wilk's test [35] for normality and Barlett's test for homogeneity of variances were used. The non-parametric alternative, Friedman's method for randomized blocks was used to first determine if there was a difference among the groups, then in the cases where a null hypothesis of no difference was rejected, a *post hoc *analysis was subsequently used. All tests were carried out using the statistical language R [36] and an implementation of the *post hoc *test was written in R and based on the coin package [37].

### Application to gene-expression data

To illustrate the utility of our proposed method, we applied the algorithm to time-series microarray data [13]. The *K*-means clustering algorithm [38] was used and all clusters that contained one or more genes annotated with a GO term pertaining to 'mitochondria' were used to create a gene set of interest from the original probes. In all, the gene set of interest contained 458 genes. Next, spectral clustering was run on the gene set using GO, publications, gene expression, and all possible combinations of the individual sources. For the gene expression data the correlation coefficient was used as a distance metric. The purpose of the experiment was to determine if our approach is capable of identifying coherent subsets among these genes by different combinations of information data sources as a means to reveal new biological information. In addition, we were interested in the relative performance of each information source *k *so in order to ensure an unbiased comparison *k *was set to 10 for each information source used. The performance of the information sources is compared using a weighted mean of the p-values (see Figure 5). After each partitioning of the genes into putative modules each cluster was assessed for functional coherence using the GOSteiner method [18].

## Authors' contributions

AJR and XL conceived the project. AJR performed the experiments and carried out the data analyses. JHS participated in study design. BR and LAC helped with the the method application and results interpretation. AJR and XL produced the initial draft of the manuscript, and JHS, BR, and LAC also contributed to the manuscript drafting and revision process.

## Competing interests

The authors declare that they have no competing interests.

## Supplementary Material

Additional file 1 **A PDF file contains supplementary methods and results**. Click here for file
