# Supplementary material for: Revealing functionally coherent subsets using a spectral clustering and an information integration approach
Source: BMC Syst Biol. 2012 Dec 17;6(Suppl 3):S7. doi: 10.1186/1752-0509-6-S3-S7 (PMC3542577; doi:10.1186/1752-0509-6-S3-S7)
Supplement: Additional file 1 — A PDF file contains supplementary methods and results. [file 1752-0509-6-S3-S7-S1.pdf]

Revealing functionally coherent subsets using a spectral clustering and an information integration approach
Additional File

Adam J. Richards [1,4*], John H. Schwacke [1], Bärbel Rohrer [2,3]
L. Ashley Cowart [1], & Xinghua Lu [*1,5] [*]

July 29, 2012

[1]Department of Biochemistry and Molecular Biology, [2]Department of Ophthalmology, and [3]Department of Neurosciences, Medical University of South Carolina, Charleston, SC USA, [4]Department of Biostatistics and Bioinformatics, Duke University, NC USA and [5]Department of Biomedical Informatics, University of Pittsburgh, PA USA

[*]to whom correspondence should be addressed

Contents

1 Supplemental Methods **2**
1.1 Data integration and retrieval framework 2
1.2 Genomic sequence: an information source of co-regulation among genes. 2
1.3 Evaluation of clustering results 3

2 Supplemental Results **3**
2.1 DNA motifs data as an information source 3
2.2 GO Graphs by Aspect 4
2.3 Noise Filtering: An Example 4

3 Supplemental Tables **6**
3.1 Estimates of kernel bandwidth parameter 6

1 Supplemental Methods

1.1 Data integration and retrieval framework

To help maximize the reusability and flexibility of our data management system, we used the representational state transfer (REST) architectural style, which is becoming more widely used in biology [3]. An architectural style in general is defined by the configuration of architectural elements including: components, connectors, data, and the relationships among them. Under this style, the communication is carried out using *resources* which are identified by Uniform Resource Identifiers or URI's. The methods described in this work use a centralized database scheme, which contains multiple information types that may be queried through the use of resources. For the database we used PostgreSQL (http://www.postgresql.org), although the code is organized in such a way that it is essentially database agnostic. To deal with the *business logic* or exchange of information with the database, we used the SQL toolkit and object relational mapper SQLAlchemy (http://www.sqlalchemy.org). The Pylons http://pylonshq.com/ framework was used to implement a RESTful storage and retrieval system. Under this setting, a Model-View-Controller (MVC) paradigm is followed. The majority of scripting was carried out using the Python (http://www.python.org) programming language, with a few additional scripts being written in Perl (http://www.perl.org). A basic API was developed for the R statistical language [4] and for Python.

1.2 Genomic sequence: an information source of co-regulation among genes.

Functionally related genes tend to be co-regulated at the transcription level in order to interact or cooperate at the protein level; thus genes sharing genomic motifs that potentially function as transcription factor binding sites (TFBSs) are likely to be related functionally. A large number of search methods have been proposed for the discovery of TFBS, yet entropy-based methods are the ones most commonly used [7]. The putative TFBS were found by searching promoter regions using the position specific scoring matrices (PSSM) available from the TRANSFAC [2] database

based on the maximum entropy scores. The promoter regions were obtained from the NCBI contig builds available from ftp://ftp.ncbi.nih.gov/genomes/ and the regions were defined as -300bp to +1000bp relative to the transcription start site. For each PSSM, we scanned through all genes in the genome of interest and obtained a maximum entropy score (x) for each gene. These scores were compiled into empirical cdf distributions, in order to determine p-value of a PSSM instance. The one-sided p-value, as determined from the empirical cdf, can be interpreted as the chance of observing a given entropy value or a value with a better match when considering all genes in the genome. The threshold for significance was set at p-value ≤ 0.05.

1.3 Evaluation of clustering results

Given the resulting labels from the algorithm and the original labels, one of the more popular methods of evaluation is the Rand index [5]. This is a comparison by *counting pairs* and measures the extent to which clusters agree or disagree. Let a cluster $\mathcal{C} = \{C_1, \ldots, C_K\}$ be the partitions of N observations $X = \{x_1, \ldots, x_N\}$ such that $\bigcup_{k=1}^{K} C_k = X$. Let the original clustering be denoted by $\mathcal{C}$ and another clustering for the same data X be $\mathcal{C}'$. Pairs of observations may be counted in the following ways:

$$
\begin{aligned}
a &= \text{pairs that are in same cluster under both } \mathcal{C} \text{ and } \mathcal{C}' \\
b &= \text{pairs that are in same cluster under } \mathcal{C}' \text{ but not under } \mathcal{C} \\
c &= \text{pairs that are in same clusters under } \mathcal{C} \text{ but not under } \mathcal{C}' \\
d &= \text{pairs that are in different clusters under both } \mathcal{C} \text{ and } \mathcal{C}'
\end{aligned}
$$

The Rand index was originally designed to assess partition accuracy of two classes, but here we are interested in larger values of k. Therefore, we adopted the statistics from the Rand index, and used them to calculate modified recall and precision measures. The former can be defined as $\frac{a}{a+c}$ and the latter as $\frac{a}{a+b}$. A metric that combines both of these measures is the F_1 score,

$$
\text{F}_1\text{score} = 2\frac{\left(\frac{a}{a+c} \times \frac{a}{a+b}\right)}{\left(\frac{a}{a+c} + \frac{a}{a+b}\right)}. \tag{1}
$$

2 Supplemental Results

2.1 DNA motifs data as an information source

The objective in these experiments was to evaluate DNA motifs as a potential source of information in the discovery of functional modules. Distances between genes were calculated as the maximum number of shared transcription factor binding sites minus the observed number of shared transcription factor binding sites. The motif data are different from the GO and PubMed sources in that they represent more of an experimental-type data source. Simulations were run as was done with the GO and PubMed data sources, however, this time including motifs as an information source. Figure 1 demonstrates the effect of utilizing the motif data alone as an information source, and how

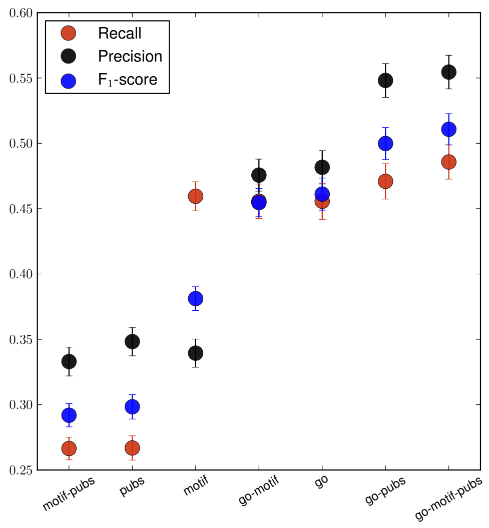

Figure 1: **Motif information integration**. The discriminative abilities as represented by recall, precision and F_1 scores, for the different source combinations. Each source represents a summary of 120 individual simulations for the species *S. cerevisiae* with k ranging from 3-8. The standard error bars are given for each discriminative measure and for each of the three species.

it compares to the GO and PubMed data sources. The results can be summarized as follows. 1) Using each information source alone, the performance of spectral clustering becomes increasingly better in the following order: PubMed, DNA motif, and the GO. 2) Combining PubMed with DNA motifs and combining GO with DNA motifs does not lead to significant enhancements, although combining GO, PubMed and DNA motifs have led to marginal enhancement in comparison with combination of GO and PubMed data.

A possible explanation for the lack of enhancement by adding the DNA motif data is that, although by itself the DNA motif information performs much better in comparison to PubMed, is that only a limited number of TFBS are experimentally characterized compared to the number thought to exist. The trend may also be explained by the degenerative nature of TFBS, which causes search methods to have high numbers of false positives [1].

2.2 GO Graphs by Aspect

The distances between genes can be measured by any of the three Gene Ontology aspects individually or more than one in combination via kernel fusion. Here we show the differences between these aspects in terms of all pairwise distances in each of the three graphs.

2.3 Noise Filtering: An Example

A major objective of the information integration and clustering framework is to direct attention to genes that, based on the given data source(s), represent the most coherent subsets. This challenge

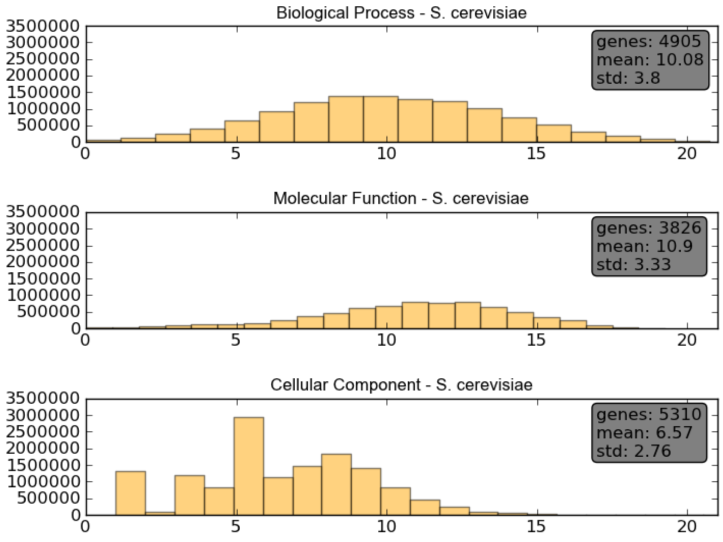

Figure 2: **GO Graphs by aspect**. Histograms of the three aspects of the Gene Ontology. The number of genes present, mean and standard deviation are shown for each. On the x-axis the semantic distance as calculated in the manuscript is shown

is compounded by the presence of noise. In the manuscript, a figure summarizes the results of a number of simulations under conditions with varying levels of noise. In Figure 3 the results of one of those simulations are broken down to illustrate the decisions made by the algorithm at the gene level.

In this experiment, we used a MINT protein interaction complex (*S. cerevisiae*) that consisted of 23 genes to which we added 27 genes randomly selected from the genome. The goal was to run the algorithm, without making an assumption for k, and check the results to see if we could recover significantly coherent subset(s) that make up the original 23 genes. Using combined GO and PubMed data as information sources, our algorithm broke the list of 50 genes into 4 clusters. Two of the clusters contained only a single gene, and could not be assessed for statistical significance. The remaining two modules contained 21 and 23 genes. After being subjected to coherence testing using the GOSteiner method [6], their respective p-values were 0.999 and < 0.0001. The sole statistically significant module contained 18/23 (i.e. 78%) of the genes from the original coherent gene set and five noise genes (false positives; 22%). In summary, our procedure identified 4/50 genes explicitly as noise and 23/50 genes were determined as noise by statistical significance testing, using GOSteiner. Overall, this is an impressive result indicating that the techniques in our procedure (spectral clustering, information integration, noise modeling, determination of cluster number, and finally functional coherence assessment) successfully revealed the majority of the truly coherent genes as a candidate subset.

To further evaluate the results, we inspected the affinity matrix representing the 50 genes. Elements of the matrix, whose values are above the 50 percentile, were included as edges connecting

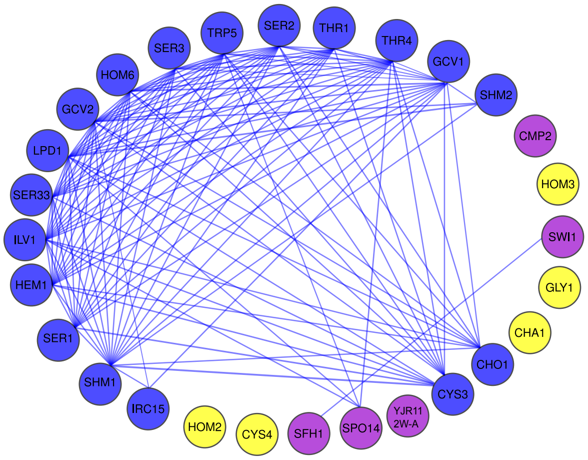

Figure 3: **A functional module (MINT) uncovered from noise**. The original gene list was comprised of 23 genes belonging to a protein complex, and 27 that were noise. Spectral clustering was run on the gene list, and statistical significance was determined for each of the underlying modules. Shown here is the one statistically significant module. Also, added in yellow are the protein complex genes that fell into other modules. The genes that were significant and truly belonged to the complex are marked as a true positive (blue). If significance was determined and the gene was truly noise then it is shown in violet (false positive), and the genes missed by the module are shown in yellow (false negative). The true negatives are removed from the graph for clarity. The edges with a weight (affinity) less than the median edge weight are also removed for ease of visualization.

the genes. The connectivity of the genes in the significant cluster were shown in Figure 3. In addition to the 23 member genes of the cluster, we also included the 5 genes from the original coherent set that were not identified to belong to the significant module (yellow nodes), to illustrate how these genes were clustered in our process. The true positive genes (blue nodes) are all highly connected to each other with strong edges; there is connectivity between three true positives, and a false positive gene (*SPO14*). From these observations, we see that the false positive genes are weakly connected to the rest of the true positive genes, which explains why they were not included as part of the noise group. The results indicate that spectral clustering is capable of accurately capturing the relatedness of functionally coherent genes, and our overall procedure is capable of dealing with noise inevitably found in biological data.

3 Supplemental Tables

3.1 Estimates of kernel bandwidth parameter

Affinities were calculated using the Gaussian kernel function:

$$A_{ij} = \exp\left(\frac{-||d_{ij}||^2}{2\sigma^2}\right), \tag{2}$$

	Saccharomyces cerevisiae	*Mus musculus*	*Homo sapiens*
GO	0.3	2.0	0.3
Publications	0.0015	4.0	0.001
Motif	0.5	0.5	0.5

Table 1: Estimated values for the bandwith parameter σ.

where $||d_{ij}||$ is a measure of distance between objects i and j and σ is the bandwidth parameter. In Table 3.1 the estimates, by species and information source, are reported.

References

[1] J Hu, B Li, and D Kihara. Limitations and potentials of current motif discovery algorithms. *Nucleic Acids Research*, 33(15):4899–4913, 2005.

[2] V Matys, E Fricke, R Geffers, E Gössling, M Haubrock, R Hehl, K Hornischer, D Karas, A E Kel, O V Kel-Margoulis, D-U Kloos, S Land, B Lewicki-Potapov, H Michael, R Münch, I Reuter, S Rotert, H Saxel, M Scheer, S Thiele, and E Wingender. TRANSFAC: transcriptional regulation, from patterns to profiles. *Nucleic Acids Research*, 31(1):374–378, Jan 2003.

[3] H Mcwilliam, F Valentin, M Goujon, W Li, M Narayanasamy, J Martin, T Miyar, and R Lopez. Web services at the European Bioinformatics institute-2009. *Nucleic Acids Research*, 37(Web Server issue):W6–W10, Jul 2009.

[4] R Development Core Team. *R: a language and environment for statistical computing*. R Foundation for Statistical Computing, Vienna, Austria, 2008. ISBN 3-900051-07-0.

[5] W M Rand. Objective criteria for the evaluation of clustering methods. *Journal of the American Statistical Association*, 66(336):846–850, 1971.

[6] A J Richards, B Muller, M Shotwell, L A Cowart, B Rohrer, and X Lu. Assessing the functional coherence of gene sets with metrics based on the Gene Ontology graph. *Bioinformatics*, 26(12):i79–i87, Jun 2010.

[7] G D Stormo. DNA binding sites: representation and discovery. *Bioinformatics*, 16(1):16–23, Jan 2000.
